# Prediction of response to PPI therapy and factors influencing treatment outcome in patients with GORD: a prospective pragmatic trial using pantoprazole

**DOI:** 10.1186/1471-230X-11-52

**Published:** 2011-05-11

**Authors:** Robert C Heading, Hubert Mönnikes, Anne Tholen, Holger Schmitt

**Affiliations:** 1 Retired Gastroenterologist, Edinburgh, UK; 2 Department of Medicine, Martin-Luther Hospital, Academic Teaching Hospital of Charité Universitätsmedizin, Berlin, Germany; 3 Nycomed International Management GmbH, Zurich, Switzerland; 4 Nycomed GmbH, Constance, Germany

## Abstract

**Background:**

Management of patients with gastro-oesophageal reflux disease (GORD) can be assisted by information predicting the likely response to proton pump inhibitor (PPI) treatment. The aim was to undertake a study of GORD patients designed to approximate ordinary clinical practice that would identify patient characteristics predicting symptomatic response to pantoprazole treatment.

**Methods:**

1888 patients with symptoms of GORD were enrolled in a multicentre, multinational, prospective, open study of 8 weeks pantoprazole treatment, 40 mg daily. Response was assessed by using the ReQuest™ questionnaire, by the investigator making conventional clinical enquiry and by asking patients about their satisfaction with symptom control. Factors including pre-treatment oesophagitis, gender, age, body mass index (BMI), *Helicobacter pylori *status, anxiety and depression, and concurrent IBS symptoms were examined using logistic regression to determine if they were related to response, judged from the ReQuest™-GI score.

**Results:**

Poorer treatment responses were associated with non-erosive reflux disease, female gender, lower BMI, anxiety and concurrent irritable bowel syndrome symptoms before treatment. No association was found with age, *Helicobacter pylori *status or oesophagitis grade. Some reflux-related symptoms were still present in 14% of patients who declared themselves 'well-satisfied' with their symptom control.

**Conclusions:**

Some readily identifiable features help to predict symptomatic responses to a PPI and consequently may help in managing patient expectation. ClinicalTrial.gov identifier: NCT00312806.

## Background

Despite the potency of proton pump inhibitors (PPIs) on gastric acid secretion, it is now evident that they do not suppress symptoms in patients with gastro-oesophageal reflux disease (GORD) as completely as was once supposed. Indeed, with the advent of more thorough symptom evaluation, it is now well recognised that both in the clinical study setting and in ordinary clinical practice, many GORD patients experience persistence of troublesome reflux symptoms while taking PPI therapy[1-3]. Thus, physicians prescribing a PPI for GORD have an obligation to assess the patient's symptoms at an appropriate time after starting treatment and to do so without any preconceived belief that the PPIs are invariably successful.

Patients with classical gastro-oesophageal reflux symptoms often experience other troublesome symptoms, such as sleep disturbance, and gastro-intestinal (GI) symptoms reflective of irritable bowel syndrome (IBS)[4-7]. The overall symptom burden results from the summation of all these symptoms and therapeutic studies that adopt a narrow focus on the classic symptoms, such as heartburn, risk failing to relate to the more complex symptom burden that is the patient's experience. Partly for this reason, systematic questionnaires (such as ReQuest™) have been developed to quantify the broader range of symptoms shown to be GORD-related[8,9].

To investigate issues of practical concern to physicians managing GORD patients in an ordinary practice setting, particularly to address a question that physicians often ask themselves when initiating PPI treatment for GORD, namely 'will this patient respond well or poorly to treatment?', we have examined features that might help to predict the treatment response.

## Methods

### Patients and design

This was a multicentre, multinational (167 centres in 21 countries), prospective, open study (ClinicalTrial.gov identifier: NCT00312806). A pragmatic (as opposed to an explanatory) design was adopted so that the conditions of the study would resemble the conditions of ordinary clinical practice so far as possible, thereby optimising the likelihood of the results being relevant to everyday practice [10,11]. Thus patients were enrolled on the basis of having symptoms considered by the investigating physician to justify a diagnosis of GORD, without further specification of GORD diagnostic criteria. However, participating patients had to be adults (aged ≥18 years), able to give informed consent and to be thought likely in the investigator's judgment to comply with the requirements of the study, particularly completion of the questionnaires.

Patients with symptoms or evidence of complicated GORD, with previous upper GI surgery or who had received *Helicobacter pylori (H. pylori) *eradication treatment in the preceding 4 weeks were excluded, as were those who had recently taken acid-suppressing medications, corticosteroids, non-steroidal anti-inflammatory drugs or prokinetics. These medications together with sucralfate, misoprostol, bismuth preparations, other substances with influence on the relief of acid-related symptoms, ketoconazole and other drugs showing pH-dependent absorption were not permitted during the period of the study. The study was carried out in accordance with the Declaration of Helsinki. Ethics approval for the study was obtained locally by all participating investigation sites.

At enrolment of the patient, the investigator enquired about the presence of symptoms that would be consistent with IBS and answered 'yes', 'no', or 'I do not know' to the question: 'Is it possible that this patient does not only suffer from GORD-related symptoms, but also from symptoms caused by irritable bowel syndrome (IBS)?' This is consistent with the pragmatic design of the study, and is reflective of clinical practice in many countries where general practitioners may not be familiar with the Rome III criteria and IBS is largely diagnosed based on patient history[12,13].

After enrolment, upper GI endoscopy was performed to categorise patients as having erosive or non-erosive reflux disease (ERD or NERD) and to grade oesophagitis, if present, according to the Los Angeles classification[14,15]. Patients were excluded from further participation in the study if the endoscopy identified an oesophageal stricture, a Schatzki's ring, an oesophageal diverticulum, oesophageal varices, or Barrett's oesophagus. *H. pylori *status was determined by serology, as this is the method available to most physicians when endoscopy is not done[16].

Patients were then required to attend the investigation centre on three occasions over an 8-week period. At the first attendance (baseline, Day 0), they were given their documentation (the ReQuest™, hospital anxiety and depression scale (HADS), 'treatment satisfaction sheet' and GERDyzer™ questionnaire) and were supplied with their medication (pantoprazole 40 mg) to be commenced the following day and taken once daily before breakfast throughout the 8-week period. Consistent with a pragmatic trial design reflecting real-life clinical practice, pill counts were not conducted. While reasonable attempts are made to encourage compliance with treatment in pragmatic trial designs, these should not go beyond what is expected in normal clinical practice[17]. The first ReQuest™, HADS and GERDyzer™ questionnaires were to be completed in relation to Day 0 (i.e. the day before commencing treatment) and ReQuest™ daily thereafter. HADS and GERDyzer™ questionnaires and the treatment satisfaction sheet were also completed in relation to the day before each of the subsequent visits at Week 4 and Week 8, when they were handed in. At each of these two visits, the investigator questioned the patient to establish the 'investigator's assessment' of the adequacy of symptom control.

### Assessments

#### ReQuest™

ReQuest™ is a self-administered scale that provides a comprehensive evaluation of symptoms in patients suffering from GORD[18-20]. Both, a long and a short version have been validated in several languages: the short version was used in this study. ReQuest™ has not been validated for the assessment of quality of life. Rather, it assesses seven dimensions of GORD, namely acid complaints, upper abdominal/stomach complaints, lower abdominal/digestive complaints, nausea, sleep disturbances, other complaints, and general well-being. Each dimension is assessed in respect of intensity and frequency. Intensity is measured by a 100 mm visual analogue scale (VAS) ranging from 'not at all' to 'extremely severe', whereas frequency is measured by a 7-point Likert scale ranging from '0' to 'more than 10 times per day' or 'continuously'. General well-being is measured on a VAS, ranging from 'wonderful' to 'extremely poor'.

The dimensions of the ReQuest™ can be grouped into two sub-scales: ReQuest™-GI (gastrointestinal: includes acid complaints, upper abdominal/stomach complaints, lower abdominal/digestive complaints, and nausea) and ReQuest™-WSO (general well-being, sleep disorders, and other complaints). The ranges of the ReQuest™ and its sub-scores are as follows:

• ReQuest™ total score: 0 to 46.28,

• ReQuest™-GI: 0 to 30.77,

• ReQuest™-WSO: 0 to 15.51.

Patients were considered to have responded to treatment (to be 'responders') if their ReQuest™-GI symptom score was below 1.6 on 3 consecutive days. A score of 1.6 was the 95% upper confidence limit of the scores found in healthy subjects[21]. Although only the ReQuest™-GI sub-scale was used to define response to treatment, both sub-scales and the total score were used in exploring the potential of ReQuest™ to predict treatment outcome.

#### HADS

The HADS was used to assess relationships between the patients' symptoms and psychological constitution[22]. The HADS is a well established screening measure for anxiety and depression used in outpatient clinics. It comprises 14 items and is subdivided in two subscales with 7 items relating to anxiety and 7 to depression. The two subscales each range from 0 to 21 and the total scale from 0 to 42. According to Snaith[23], a score of 0 to 7 for either subscale can be regarded as the normal range; a score of 8 to 10 is suggestive of the presence of the respective state and a score of 11 or higher indicates the probable presence of a mood disorder.

#### Patient satisfaction

At the follow up visits to the investigation site 4 and 8 weeks after commencing treatment, patients handed in their treatment satisfaction sheet, categorising their satisfaction with symptom control during the preceding 24 hours as 'very satisfied', 'fairly satisfied' or 'not satisfied'.

#### Investigator assessment

At the same visits, the investigator made conventional clinical enquiry about the patient's symptoms and assessed them as being 'well-controlled', 'fairly-controlled' or 'not controlled'.

#### Quality of Life assessment: the GERDyzer™ questionnaire

The GERD Analyzer (GERDyzer™) is a health-related quality of life questionnaire to evaluate the impact of GORD on the patient's quality of life[24]. GERDyzer™ assesses 10 dimensions of quality of life (i.e. general well-being, pain/discomfort, physical health, energy, daily activities, leisure activities, social life, diet/eating/drinking habits, mood and sleep). Each dimension is assessed using a 100 mm VAS ranging from 'not at all' to 'very much' (except 'general well-being': from 'excellent to 'unbearably bad'): higher GERDyzer™ scores indicate greater quality of life impairment.

### Statistical analysis

The factors investigated as possible influences on response to treatment were: age, body mass index (BMI), gender, geographical location, *H. pylori *status, symptoms suggesting IBS as well as GORD, presence of oesophagitis before treatment, the grade of oesophagitis, if present, and the HADS scores (total and sub-scores). All factors were described by frequencies and percentages for the subgroups responder and non-responder. An univariate logistic regression analysis was used to assess the influence of each of these factors, with the appropriate response rates after 8 weeks treatment (proportion of patients being responders to treatment) being the dependent variable and the investigated factors as independent variables. A p-value of < 0.05 was taken to indicate significant influence of the independent variable. In addition, a global multivariate analysis was performed to take into account any effects of confounding. The regression model considered the HADS as total score and ERD/NERD only by their presence rather than by oesophagitis grades.

Some reports in the literature indicate that symptom severity before treatment and/or the symptom response occurring in the first few days of treatment can predict the later treatment outcome[25-29]. For this reason, ReQuest™ total and subscale scores relating to the pre-treatment day and the first 10 days of treatment were examined to identify the potential for the scores to serve as a clinically useful predictor of treatment outcome (response or non-response) at 8 weeks. For each of the 11 days, response and non-response prediction rates for the whole score ranges of the respective ReQuest™ scores were determined. The premise was that scores equal to or below a selected level would predict 'response' and those above another selected level would predict 'non-response'. Taking scores in increasing steps of 0.01 from 0 to the maximum score on each of the 11 days, prediction rates for response and non-response were calculated as:

For all time points, the highest prediction rates for response and non-response at the end of 8 weeks treatment were identified. The difference between the corresponding response and non-response prediction levels was then calculated and the lowest difference identified. This permitted the range of score values failing to predict either response or non-response to be as small as possible. After the determination of the ReQuest™ score, which fits the above described properties best, a logistic regression analysis (as specified above) was conducted to investigate the possible influence of this ReQuest™ score on the response to treatment. All calculated p-values were interpreted in an explorative sense [30].

## Results

In total, 1928 patients were recruited through 167 investigational sites (specialist and primary care centres) in 21 countries. The safety population comprised 1901 patients. The intention-to-treat population (ITT) was 1888 patients: their demographic and other data at baseline are presented in Tables 1 and 2.

**Table 1:** Demographic data and baseline characteristics (ITT, n = 1888)

Age [years], mean (SD)		47.0	(14.3)
Height [cm], mean (SD)		167.5	(9.6)
Weight [kg], mean (SD)		74.2	(15.8)
BMI [kg/m^2^], mean (SD)		26.4	(4.8)
Gender, n (%)	Female	978	(51.8)
	Male	910	(48.2)

Ethnic origin, n (%)	White	1326	(70.2)
	Asian	352	(18.6)
	Other	167	(8.8)
	Black	43	(2.3)

Patients per continent, n (%)	Western Europe	894	(47.4)
	South America	352	(18.6)
	Asia	326	(17.3)
	Canada	124	(6.6)
	South Africa	100	(5.3)
	Australia	92	(4.9)

Smoker, n (%)	Never	1179	(62.4)
	Former	360	(19.1)
	Current	349	(18.5)

**Table 2:** Additional baseline data (ITT, n = 1888)

		N	(%)
***H. pylori *status**	Positive	687	(36.4)
	Negative	1070	(56.7)
	Intermediate	98	(5.2)
	Missing	33	(1.7)

**Oesophagitis**	Non-erosive	694	(36.8)
	Grade A	680	(36.0)
	Grade B	381	(20.2)
	Grade C	97	(5.1)
	Grade D	23	(1.2)
	Missing	13	(0.7)

### Response rates

The overall response rates (i.e., ReQuest™-GI symptom score below 1.6 on 3 consecutive days) for the ITT population were 58.9% at Week 4 and 71.2% at Week 8.

Logistic regression analysis data assessing the influence of various factors independently on response rates following pantoprazole treatment is presented in Table 3. The presence of ERD, lower baseline HADS scores (total and sub-scores) and higher BMI were associated with a response to therapy (all p < 0.0001), as were male gender (p = 0.0011) and differences in geographic location (p = 0.0052). Concurrent IBS symptoms were associated with poorer response to treatment (p < 0.0001). In contrast, pre-treatment *H. pylori *status, oesophagitis grade and age showed no statistically significant influence on treatment response (Table 3). Further details of the factors influencing response are presented below.

**Table 3:** Factors influencing treatment response

Factor	Logistic regression p-value
ERD/NERD	<0.0001
HADS total score	<0.0001
• Anxiety sub-score	<0.0001
• Depression sub-score	<0.0001
IBS	<0.0001
BMI	<0.0001
Gender	0.0011
Geography	0.0052

*H. pylori*	Not significant
Age	Not significant
Oesophagitis grade (A-D)	Not significant

The results of the global multivariate regression analysis revealed one difference to the univariate model: for the factor gender, no significant influence on response was found. All other factors that were significantly associated with treatment response in the univariate analysis were likewise significant in the multivariate analysis.

### ERD vs. NERD; gender

**Figure 1:**
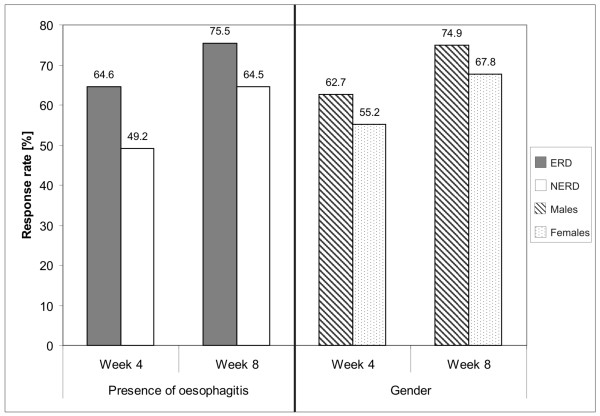
**Response rates after 4 and 8 weeks: presence of oesophagitis and gender**. Statistical results are given in Table 3 and in the text.

At the end of the 8 weeks of treatment, patients who had erosive reflux disease (ERD) pre-treatment showed higher response rates than those with NERD (75.5% vs. 64.5%; Figure 1). Among patients with ERD before treatment, however, no statistically significant differences in response rates were seen according to grade of oesophagitis. Male patients had a higher response rate than female patients (74.9% vs. 67.8%; Figure 1).

### Geography

In Western Europe, South America and Canada, response rates were comparable at Week 4 (approximately 60%) and at Week 8 (approximately 72%). Highest response rates after 8 weeks of treatment were found in South Africa (82.5%), lowest response rates in Asia (60.8%) and Australia (61.7%).

### Anxiety and depression

**Figure 2:**
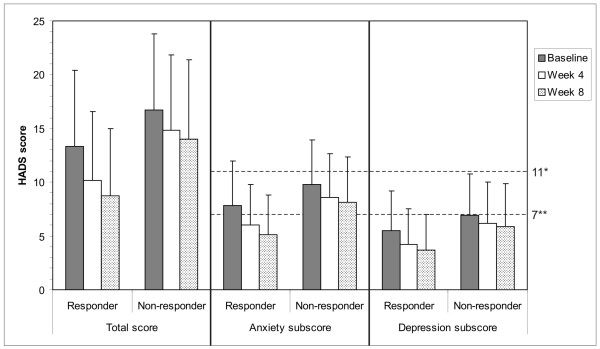
**HADS scores**. Statistical results are given in Table 3 and in the text. *Score of ≥11: probable presence of mood disorder. **Score of ≥7 to <11: suggestive for presence of mood disorder (scores <7: normal range).

The HADS total score and both sub-scores (Figure 2) decreased over time for responders as well as for non-responders. At baseline, both, responders and non-responders had a mean anxiety sub-score suggestive of the presence of an anxiety state. Under treatment, the score fell to normal in the responders (below 7; Figure 2). A fall was also apparent in non-responders, but their score was still above normal at Week 8. In contrast, the depression sub-scores provided no evidence of depression at any time in either responders or non-responders (all scores were below 7 at all timepoints; Figure 2). Overall, non-responders had higher HADS scores (total and sub-scores) than responders at all estimated time points. Higher baseline HADS scores (total and both sub-scores) were thus statistically associated with non-response to treatment.

### Irritable bowel syndrome

**Figure 3:**
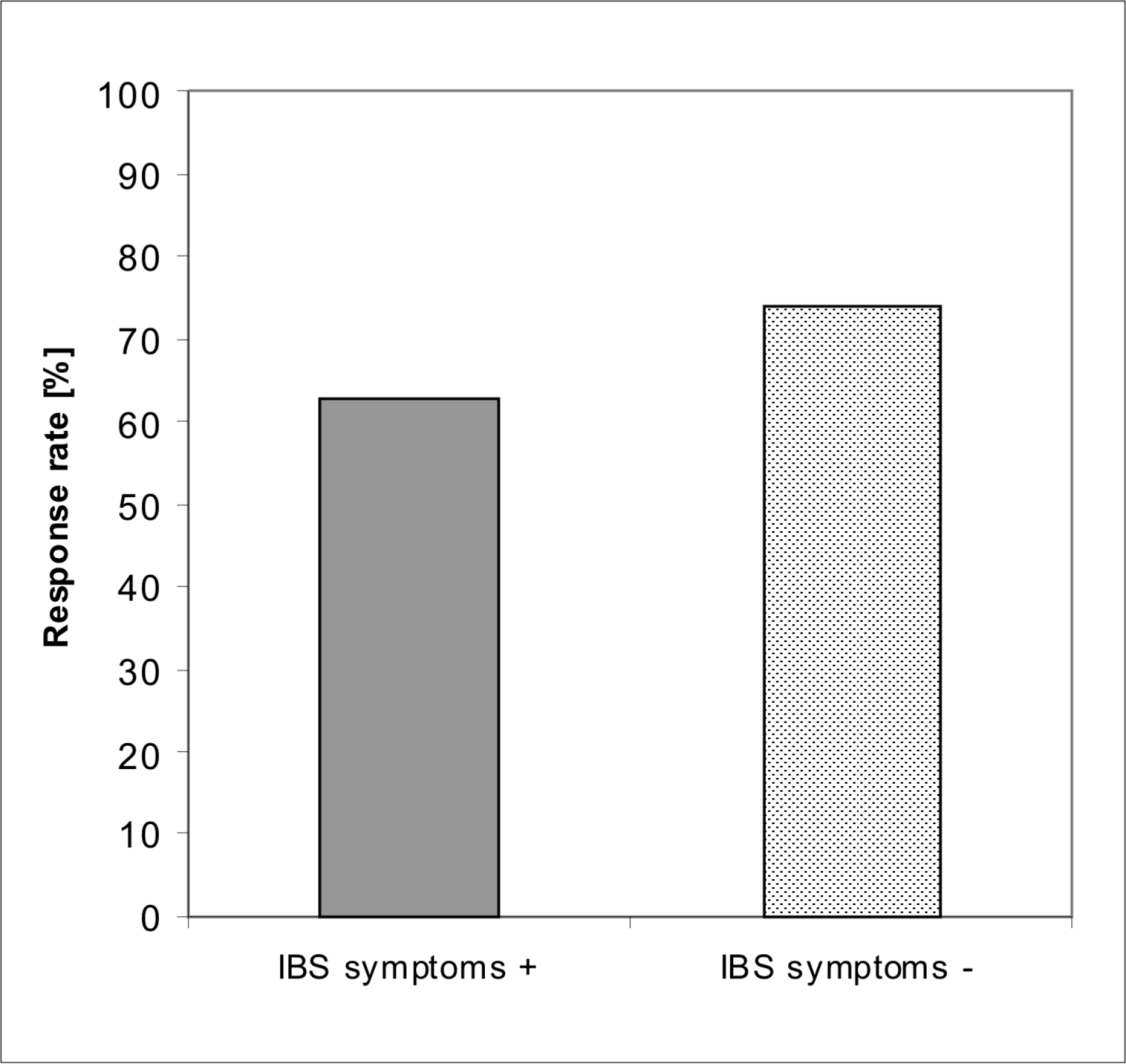
**Response rates at 8 weeks according to the presence or absence of IBS symptoms at baseline**. Statistical results are given in Table 3 and in the text.

Symptoms suggesting concurrent IBS at the time GORD treatment was begun were found to be associated with poorer response rates. The findings at 8 weeks are shown in Figure 3.

### Body Mass Index

BMI was positively associated with response to treatment (Table 3). Differences were found between responders and non-responders both at Week 4 (BMI = 26.9 vs. 25.7 respectively) and at Week 8 (BMI = 26.8 vs. 25.7).

### Prediction of response using ReQuest™ scores

Table 4 shows the optimum prediction levels and the corresponding prediction rates for response and non-response (i.e. the proportion of patients with a score below/above the respective prediction level who were classified as responders/non-responders to treatment at Week 8) using the ReQuest™ total score and sub-scores. The table shows, for example, that the calculated time point for the best prediction using ReQuest™-GI was Day 10. Here, 1043 patients had ReQuest™-GI scores at or below 1.47. Of these, 914 were responders at Week 8, giving a prediction rate for response of 87.6%. Of 154 patients with ReQuest™-GI scores above 8.38, 108 patients were non-responders at Week 8. Thus, the prediction rate for non-response was 69.9%. The calculated best time point of prediction for the ReQuest™ total score was also Day 10, whereas it was Day 4 for ReQuest™-WSO. Prediction rates for response and non-response were similar for ReQuest™ total score and ReQuest™-GI (response: 87%, non-response: 70%) but were lower for ReQuest™-WSO.

**Table 4:** Prediction level and prediction rates for response at Week 8

		Prediction level		Prediction rate for	
	**Time point of prediction**	**Response** (ReQuest™ score [N*])	**Non-response** (ReQuest™ score [N**])	**Response (% [N])**	**Non-response (% [N])**

**ReQuest™ total**	Day 10	<3.42 [1046]	>13.37 [148]	87.1 [911]	70.3 [104]
**ReQuest™-GI**	Day 10	<1.47 [1043]	>8.38 [154]	87.6 [914]	69.9 [108]
**ReQuest™-WSO**	Day 4	<2.24 [1057]	>4.89 [220]	82.3 [870]	60.5 [133]

**ReQuest™ total**	Baseline(Day 0)	<9.13 [1068]	>29.71 [35]	79.8 [852]	60.0 [21]
**ReQuest™-GI**		<5.91 [1047]	>21.90 [31]	80.9 [847]	61.3 [19]
**ReQuest™-WSO**		<3.10 [1044]	>11.65 [19]	80.6 [841]	73.7 [14]

Data for ITT population without missing values for response at Week 8 (n = 1738)

*Number of patients with ReQuest™ score below the prediction level of response

**Number of patients with ReQuest™ score above the prediction level of non-response

**Figure 4:**
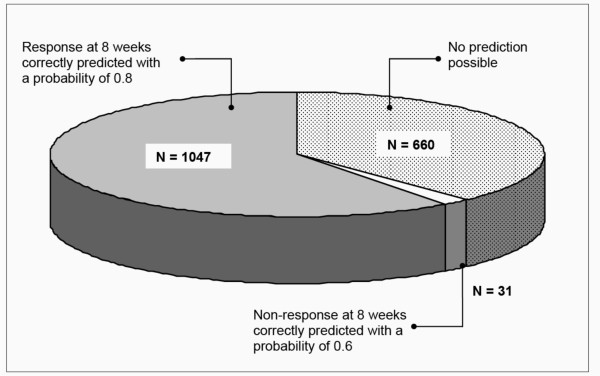
**Prediction of response using ReQuest™-GI**. N = number of patients.

In addition to identifying the time point for the best prediction, the capability of ReQuest™ scores at baseline (the day before treatment commenced) to predict treatment outcomes at Week 8 was examined and the results are also given in Table 4. The prediction rates for response were 79.8% for ReQuest™ total score, 80.9% for ReQuest™-GI, and 80.6% for ReQuest™-WSO. The prediction rates for non-response were 60.0% for ReQuest™ total score, 61.3% for ReQuest™-GI, and 73.7% for ReQuest™-WSO. Figure 4 shows these results for ReQuest™-GI in relation to the numbers of patients in whom prediction may be made.

### Control of Symptoms and Patient Satisfaction

**Figure 5:**
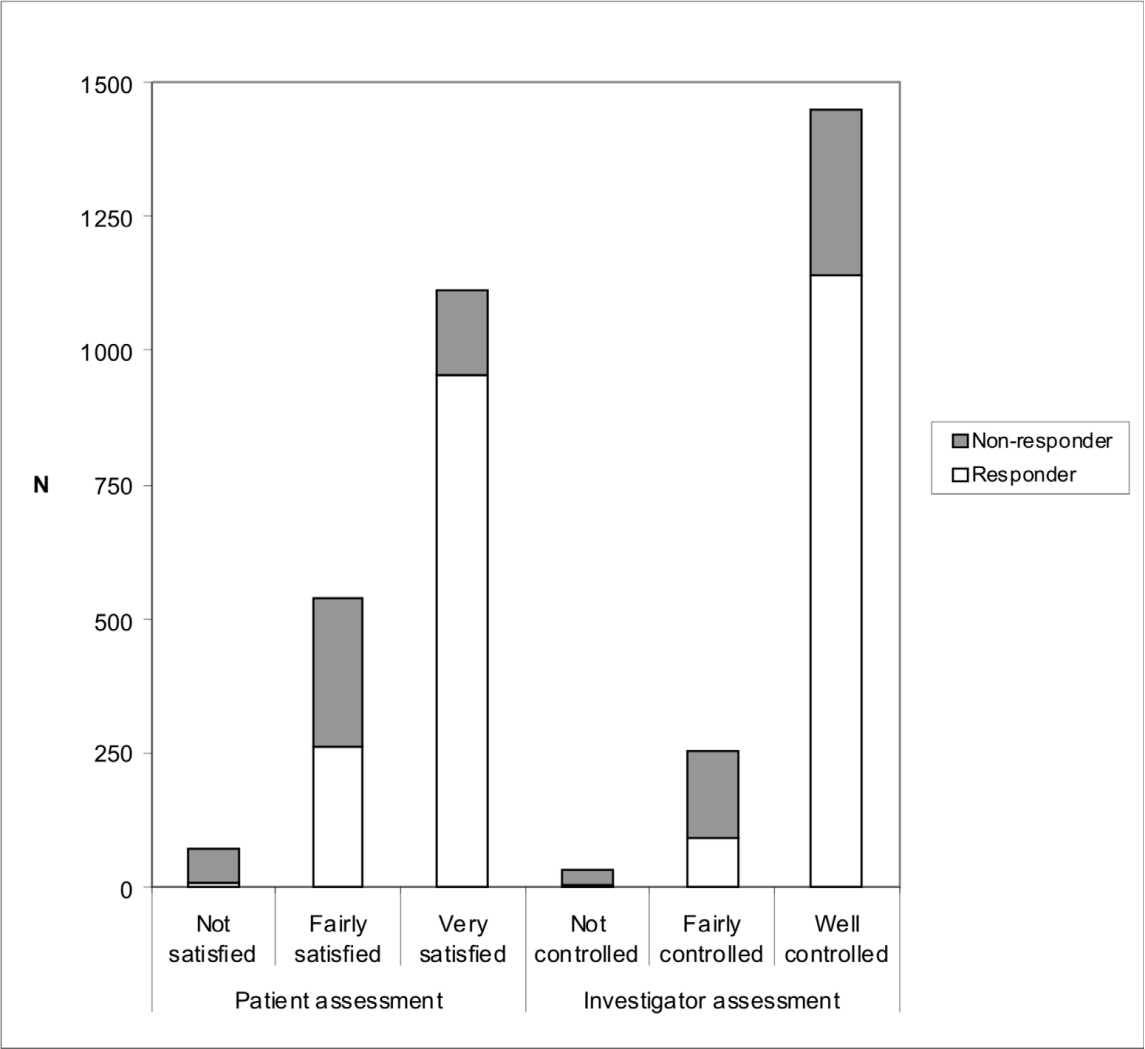
**Patient and investigator assessments at Week 8**. N = number of patients.

The investigators' assessments of patient symptom control and the patients' own assessments of their satisfaction with symptom control at Week 8 are shown in Figure 5 in relation to their categorisation as responders or non-responders. A minority of patients judged by the investigator to have their symptoms well-controlled were in fact non-responders according to ReQuest™-GI (310 out of 1450), which suggests that the investigators tended to be overoptimistic about the adequacy of symptom control achieved. Interestingly, there was also a minority of patients, albeit a smaller proportion, who were non-responders according to their ReQuest™-GI questionnaires despite declaring themselves to be very satisfied by their symptom control (155 of the 1110 who were 'very satisfied'). However, only 0.8% of patients who were responders according to ReQuest™-GI were 'not satisfied' with their symptom control.

### Quality of Life: the GERDyzer results

**Figure 6:**
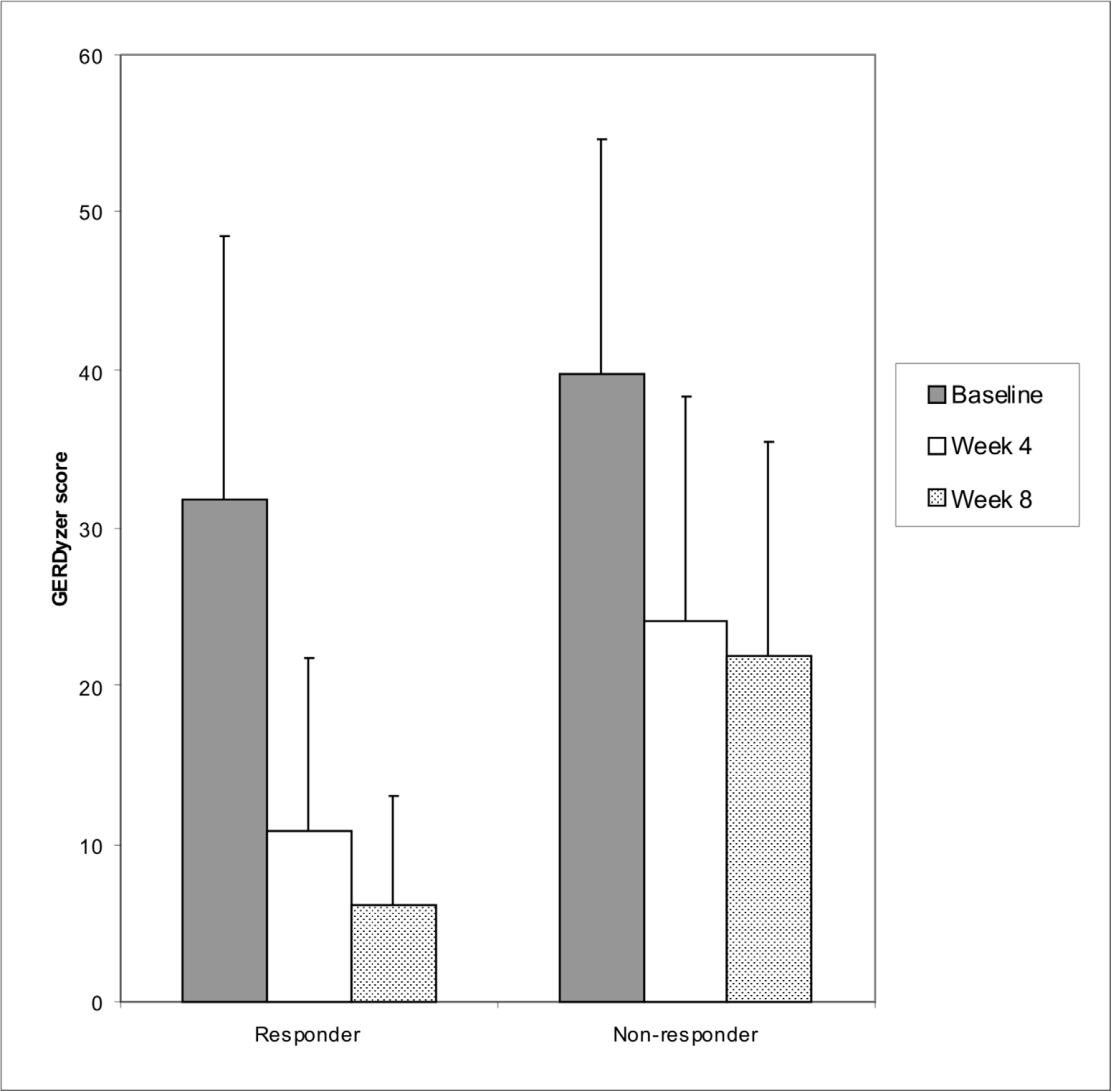
**Quality of life (GERDyzer™ scores)**. Statistical results are given in Table 3 and in the text.

The GERDyzer™ scores are shown in Figure 6. Improvement in quality of life (reduction in score) was evident during the period of treatment in both responders and non-responders, though the baseline score was higher in the non-responding group and the falls in score during treatment (baseline to Week 4 and baseline to Week 8) were significantly greater in the responders (Week 4 and Week 8: p < 0.0001). Thus more complete symptom control, as assessed by ReQuest™-GI, was associated with better quality of life.

### Adverse events

In the course of the study, 477 patients of the safety set (25.1%) experienced 784 treatment-emergent adverse events. Only 2 of these (0.3%) were assessed as definitely related to the study medication. Most of the 784 events were of mild (56.9%) or moderate (37.9%) intensity. No patient died during the study. Sixteen serious adverse events were documented in 14 patients (0.7%): 14 were assessed as unrelated and 2 as unlikely related to the intake of pantoprazole. The most frequently reported events using the terminology of the Medical Dictionary for Regulator Activities (MedDRA) were headache, diarrhoea, nausea, abdominal pain, and influenza. Other events occurred in less than 1% of the patients. Overall, the treatment was well tolerated.

## Discussion

The interpretation of clinical studies undertaken to investigate the efficacy of therapy is often constrained by the fact that the typical study setting differs from conditions in ordinary clinical practice. GORD studies undertaken on patients with oesophagitis which have used 'relief of heartburn' as the principal symptom outcome fail to take into account two important facts now widely acknowledged: first, that many GORD patients do not have oesophagitis and second, that the symptom burden experienced by GORD patients is much more complex than heartburn alone[31]. In addition, despite differences in healthcare systems it is now generally accepted that the use of PPIs to treat GORD is not conditional on first obtaining an upper GI endoscopy. The diagnosis is usually made and treatment started on the basis of clinical history, very often in a primary care setting.

To obtain information relevant to this clinical situation, our study was therefore undertaken with a pragmatic rather than explanatory trial design and patients were enrolled in the trial on the basis of a clinical history of GORD. In explanatory trials, participating patients are typically enrolled on the basis of precisely defined symptom criteria, perhaps with positive results from investigations, rather than on the basis of the way diagnoses are made in everyday clinical practice. The relevance of findings made in explanatory trials to everyday clinical practice may therefore be impaired because the patient population being studied is not the population being treated in ordinary practice[10,11]. Classical explanatory trials of PPI therapy in GORD have mostly overestimated the success of treatment in comparison with the therapeutic outcomes achieved in ordinary clinical practice[32].

A diagnosis of GORD made on the basis of the clinical history has specificity of around 65%[33,34], implying that misdiagnosis is not infrequent and this will certainly be responsible for some instances of poor treatment responses, sometimes called 'PPI failure'[35,36]. Our focus in this study was not to identify causes of PPI failure, but rather to address questions that are often at the forefront of a physician's mind when initiating PPI therapy for GORD. We selected features identifiable at the time of starting treatment that would possibly have influence on therapeutic outcome and then prospectively tested whether they did so. These features were: age, body mass index (BMI), gender, geographical location, *H. pylori *status (determined serologically), symptoms suggesting IBS as well as GORD, presence of oesophagitis before treatment, the grade of oesophagitis, if present, and anxiety and depression. Response to treatment was defined in terms of symptom relief, because in clinical practice it is not usual to perform endoscopy to assess healing of any pre-treatment oesophagitis unless troublesome symptoms persist on treatment or alarm features appear.

The patient self-administered ReQuest™ questionnaire used in this study is a validated, systematic and comprehensive assessment of frequency and severity of symptoms shown to be GORD-related[18-20]. Response to treatment is defined as a decrease in symptom score to one equal to or below the score that has been identified in healthy individuals[21].

Patients with NERD have been found in previous studies to respond to PPI treatment less well than those with ERD[37-39]. This is usually attributed to inclusion of some patients in the NERD group who do not have reflux disease, notably patients with 'functional heartburn', who are expected to respond poorly to acid suppression. In line with these findings, our data show a better response rate in patients with ERD than in NERD. In addition, our patients with ERD showed no significant differences in response rates according to grade of oesophagitis. If NERD patients with 'true' reflux disease were to respond similarly to those with ERD, these observations suggest a functional heartburn prevalence of around 4% among the patients recruited to this study.

Previously published studies are inconsistent regarding the existence of a gender difference in response to PPI treatment[26,39]. Our findings in the univariate analysis indicate that males show a better response than females. Of course, NERD is more common in females and so the lower response rates illustrated in Figure 1 may be partly explained by confounding. However, as described above, the p-values shown in Table 3 were determined from logistic regression and relate to each factor considered independently.

IBS is known to be more frequent in patients with GORD than in control populations[40-42] and is also more prevalent in females[43-45]. In our patients, concurrent IBS symptoms were associated with a lower response rate to the PPI when compared with patients without concurrent IBS symptoms. There are uncertainties about the interpretation of this observation, however. It may be supposed that the inclusion of lower abdominal complaints in the spectrum of symptoms assessed by the ReQuest™-GI questionnaire will inevitably imply a higher rate of symptom 'non-response' if these lower abdominal symptoms are unaffected by acid suppression. Surprisingly, though, previous studies using ReQuest™ have found that the lower abdominal symptoms in GORD patients do improve during PPI treatment[20,46-48] suggesting that these symptoms are not independent. Perhaps the mechanisms underlying classical reflux symptoms and the lower abdominal symptoms are somehow linked or the symptoms are linked within the patients' perceptions of overall symptom burden.

Many physicians would predict that patients with concurrent anxiety or depression would respond poorly to GORD treatment and previous studies have borne this out[26,49]. Our data likewise show that a high HADS score before treatment was associated with a poorer response, although anxiety seemed to be more relevant than depression. Nevertheless, the anxiety sub-score fell to normal in the patients who responded to treatment, consistent with a contention that GORD symptoms were causing some of the anxiety while the higher anxiety sub-scores seen in non-responders than responders, especially at baseline, are consistent with the possibility that anxiety may itself contribute to the perceived GI symptom burden. The possibility thus exists of a two way relationship between anxiety and GORD symptoms.

Our data showing an association between BMI and response rate was statistically highly significant and this observation was not expected. Although published data clearly link obesity and GORD[50-52], and indeed a correlation between BMI and GORD is also evident within the normal BMI range[53], there has been little investigation of the effect of BMI on responses to antisecretory treatment[54,55]. However, one recent study found a positive correlation between BMI and symptom relief when lansoprazole therapy was given to patients with a wide range of upper GI symptoms and negative upper GI endoscopy[56]. The authors suggested their findings could be explained by an association of higher BMI with GORD and a good response of acid reflux symptoms to treatment; this explanation could be relevant to our findings also.

An aspect of our results worth comment was the failure to find any link between age or *H. pylori *status on response rates. The literature is inconclusive with respect to *H. pylori*: Hatlebakk et al.[57] found *H. pylori *infection to have no effect on response to treatment whereas Labenz et al.[58] found greater age and a positive *H. pylori *status were both positively associated with better resolution of heartburn over a 4-week treatment period. The failure to find a link between *H. pylori *status and response may have resulted from the use of the serology for *H. pylori *diagnosis, which does not distinguish between cured and active infection[16].

Impaired quality of life is well-established as a consequence of GORD and improves with treatment[59-61]. Our results show that the baseline GERDyzer™ score was higher (indicating greater quality of life impairment) in the patients who subsequently responded poorly to treatment and that these non-responders also showed a smaller absolute improvement (fall in score) during treatment.

It is interesting to compare the symptom control determined by the ReQuest™-GI questionnaire with the investigators' assessments of symptom control and with the patients' own assessments of satisfaction with symptom control. The biggest disparity, as shown in Figure 5, was in the proportion of patients judged by the physician to have been 'well-controlled' who nevertheless were non-responders according to ReQuest™-GI. This suggests physicians tend to overestimate patients' responses to treatment, as has been previously reported[62,63]. The 'satisfaction' findings are also of interest, however, in that some 14% of patients who rated themselves to be 'very satisfied' with symptom control were 'non-responders' according to ReQuest™-GI. Patients may thus be satisfied with symptom control that is less than total, which raises an issue about whether attainment of a predefined level of symptom suppression or a 'very-satisfied' patient should be the objective of therapy. It may certainly be argued that GORD has been adequately treated if the residual symptoms a patient still has while on treatment are judged by that patient not to be troublesome. Moreover, in ordinary clinical practice it is to be expected that a patient's satisfaction with symptom control will influence the physician's assessment: a very satisfied patient may reasonably lead the doctor to consider that the GORD symptoms are 'well-controlled'. Nevertheless, the discrepancies between these different measures of treatment success illustrate the difficulties of measuring the occurrence and impact of symptoms and of establishing unequivocally what the most clinically relevant treatment goal should be. Of course, the dilemma is well recognised in other areas of clinical medicine also[64].

Geographical and ethnic differences in the nature, prevalence and presentation of GORD are recognised[65] but less is known about possible differences in response to treatment. Unfortunately, it is not clear whether the geographical variation in responses is a reflection of fundamental differences in GORD biology or an artefact of the study. Minor differences in local systems of patient recruitment, for example, may have had an effect on patient outcomes. Consequently, we do not wish to suggest any interpretation of the observed geographical variation.

Prompted by reports that treatment outcome may be predicted by symptom severity before or shortly after commencing treatment [25-29], the potential for the ReQuest™ questionnaire to predict the response to treatment was comprehensively evaluated and the results show that some predictive capability is possible both in respect of response and non-response (Table 4). The results that relate to the baseline are especially interesting in that a prediction of treatment outcome before the treatment begins could potentially help the physician in managing patient expectation. However, the probabilities of correct prediction shown in Figure 4 are perhaps lower than physicians would wish and the proportion of patients for whom no prediction could be made was higher. However, using an abbreviated form of ReQuest™, (ReQuest in Practice™)[66], scores obtained from the questionnaire were more accurate than the physicians' conventional clinical enquiry in identifying patients whose symptoms would still be controlled after stepping down from full dose to half dose PPI[67]. In this context at least, therefore, prediction based on the systematic assessment of symptom burden is potentially valuable, being more reliable than ordinary clinical judgment.

## Conclusions

Overall, these results suggest that in the setting of everyday clinical practice, several readily identifiable features can help physicians to foresee the likely success of pantoprazole treatment in controlling symptoms in GORD patients and so to manage patient expectations accordingly. While some of the observations, such as the significance of NERD rather than ERD and of concurrent IBS or anxiety are not unexpected, others such as the apparent influence of BMI and the lack of influence of oesophagitis severity might be thought contrary to clinical intuition. Of course, misdiagnosis almost certainly explains some instances of non-response to PPI treatment and the fallibility of symptom-based diagnosis of GORD is well documented. Pre-treatment identification of features that predict a poor outcome might therefore prompt the physician to review his symptom-based diagnosis of GORD before prescribing medication and consider whether any diagnostic procedures such as endoscopy or pH-metry should be undertaken.

Additionally, our results show that although there is a broad concordance between control of reflux symptoms and patient satisfaction with treatment, there are some patients who are very satisfied with their treatment despite incomplete symptom control. This serves as a reminder of the subjective nature of symptoms and of symptom impact and that patient satisfaction deserves as much consideration as direct symptom assessment when evaluating treatment success.

## Competing interests

RCH has served as a speaker, a consultant and an advisory board member for Nycomed GmbH (former ALTANA Pharma) and for Reckitt Benckiser. He owns ordinary shares in Novartis, Reckitt Benckiser and Proctor & Gamble.

HM served as a speaker, a consultant and an advisory board member for Nycomed GmbH (formerly ALTANA Pharma), Reckitt Benckiser, Steigerwald, Novartis and Movetis.

HS and AT are employees of Nycomed GmbH and Nycomed International Management GmbH.

## Authors' contributions

RCH, HM, AT and HS were all involved in the study concept and design, analysis and interpretation of data, and the critical revision of the manuscript for important intellectual content; all provided approval of the final manuscript. Additionally, RCH took a lead role in drafting of the manuscript. HS provided administrative, technical, and material support, and were responsible for study supervision.

## Pre-publication history

The pre-publication history for this paper can be accessed here:

http://www.biomedcentral.com/1471-230X/11/52/prepub
